# Resource Management Techniques for Cloud/Fog and Edge Computing: An Evaluation Framework and Classification

**DOI:** 10.3390/s21051832

**Published:** 2021-03-05

**Authors:** Adriana Mijuskovic, Alessandro Chiumento, Rob Bemthuis, Adina Aldea, Paul Havinga

**Affiliations:** 1 Department of Pervasive Systems, University of Twente, 7522 NB Enschede, The Netherlands; a.chiumento@utwente.nl (A.C.); r.h.bemthuis@utwente.nl (R.B.); p.j.m.havinga@utwente.nl (P.H.); 2 Department of Industrial Engineering and Business Information Systems, University of Twente, 7522 NB Enschede, The Netherlands; a.i.aldea@utwente.nl

**Keywords:** resource management, cloud computing, fog computing, edge computing, algorithm classification, evaluation framework

## Abstract

Processing IoT applications directly in the cloud may not be the most efficient solution for each IoT scenario, especially for time-sensitive applications. A promising alternative is to use fog and edge computing, which address the issue of managing the large data bandwidth needed by end devices. These paradigms impose to process the large amounts of generated data close to the data sources rather than in the cloud. One of the considerations of cloud-based IoT environments is resource management, which typically revolves around resource allocation, workload balance, resource provisioning, task scheduling, and QoS to achieve performance improvements. In this paper, we review resource management techniques that can be applied for cloud, fog, and edge computing. The goal of this review is to provide an evaluation framework of metrics for resource management algorithms aiming at the cloud/fog and edge environments. To this end, we first address research challenges on resource management techniques in that domain. Consequently, we classify current research contributions to support in conducting an evaluation framework. One of the main contributions is an overview and analysis of research papers addressing resource management techniques. Concluding, this review highlights opportunities of using resource management techniques within the cloud/fog/edge paradigm. This practice is still at early development and barriers need to be overcome.

## 1. Introduction

The Internet of Things (IoT) connects everyday devices with each other and with the larger Internet to bring more meaningful interactions between objects and people. The connection process typically brings together sensing, actuating, and control devices. Additionally, these devices conform to the necessary standard compliant communication protocols. IoT can realize the purpose of smart identifying, discovering, following, and controlling things in many efficient and diverse ways [1].

Thus, IoT is becoming popular in domains such as smart healthcare, transport, logistics, retail, industrial automation, and many others. For example, airports can operate in a significantly smarter manner. IoT can monitor the volume and flow of people at the airport. It can be applied in smart airfield lighting systems, to provide preventive maintenance and reduction of fuel consumption [2]. Additionally, improvement of the airport luggage delivery system can be completed by placing RFID tags and making use of smart sensors. That can be done to detect whether the luggage is transported to the proper person at the correct time and place [3]. These are a few instances that represent how the IoT technology can make the operational structure at the airport more efficient.

There are many other domains such as road and bridge monitoring, supply chain, healthcare, and water pipe monitoring, where IoT can be applied to improve the reliability of the specific information management systems.

The number of ubiquitous devices deployed in a geo-distributed manner is increasing at a rapid rate, and it is reaching up to billions. Smart devices produce an extensive amount of data, which needs to go through network infrastructures. Frequently, this can emerge as a problem. The generated data can be used to reinforce the working and evolution of smart environments. In existing cloud infrastructures, the data are sent to cloud servers for further processing and then returned to the devices. To this end, cloud computing has emerged, yet this paradigm is still commonly perceived as being at an exploratory phase. The National Institute of Standards and Technology (NIST) defines cloud computing as a design that allows sharing of many computing assets in format of services to clients. With this concept, users can efficiently modify their requirements at a low cost [4]. Another definition in a wider perspective [4] declares that services are provided by applications and systems’ software in a data center.

However, cloud computing has certain limitations: the need to transport data from each single sensor to a data center over a network, process these data, and then send instructions to actuators. This represents a large limitation because: (a) the communication increases latency considerably; and (b) since sensors and actuators are often on the same physical device, control information might be outdated as well.

Fog and edge computing may aid cloud computing in overcoming these limitations. Fog computing and edge computing are no substitutes for cloud computing as they do not completely replace it. Oppositely, the three technologies can work together to grant improved latency, reliability, and faster response times. The geo-distributed nature of the fog layer and the edge devices also enable location awareness (see the next paragraphs). One of the key differences between fog and edge computing refers to where the intelligence and the processing power reside.

Fog computing employs many nodes between the cloud and the end devices in which intelligence can be located. These allocated smart nodes represent base stations or access points [5]. By bringing intelligence away from the cloud, fog computing can process the IoT data in close proximity to the data sources. Afterward, it can use resources from the cloud (only if needed) in a more effective mode than through individual devices. For instance, fog computing can move the intelligence to a Local Area Network (LAN) position in the network architecture and thereby provide support of data processing in a fog node or an IoT gateway [6].

Edge computing is about moving the intelligence, computing power, and intercommunication capabilities of an edge gateway straight to the devices. It typically does not associate with any types of cloud-based services but concentrates more on the IoT device-side [6]. An example includes mobile services, which need ultra-low latency and real-time access to a radio network. Edge computing can be seen as an approach to forward the computation and communication resources from the cloud towards the edge. That is done to enable services by avoiding latency and thereby provide swift message delivery to users [7].

In this paper, we focus on resource management techniques for cloud, fog, and edge environments. A considerable amount of research has been done on different techniques for resource management in cloud, fog, and edge computing. A proper resource management is important because task offloading can cause more expenses in terms of downtime and energy costs (e.g., due to required communication between sensing devices and servers). Furthermore, processing excessive resources at the servers can impact the task performance delay in a system that contains a vast number of users. Hence, efficient computational offloading is relevant when dealing with IoT resource management.

Studies already provided a classification of resource management algorithms and exploratory comparative analyses of applied algorithms in the cloud, fog, or edge scenarios. However, to our knowledge, limited literature exists on analyzing resource management techniques for cloud, fog, and edge computing while taking into account resource management metrics. Some of these metrics are: resource allocation, workload balancing, resource provisioning, and task scheduling.

In this paper, we aim to present an evaluation framework for applied algorithms for resource management focusing on cloud, fog, and edge computing. It can be useful to provide researchers and practitioners insights into how resource management techniques are used within the realm of cloud, fog, and edge computing. First, analyzing existing approaches can shed some light on the current state-of-the-art and act as a source of reference for future work. Second, presenting an overview of the studied algorithms attributes and characteristics can make it possible to: (1) identify specialized solutions tailored to specific user needs; and (2) generalize about the dispersed view on the cloud, fog, and edge computing paradigm.

To provide an answer to this challenge, we first address current challenges in cloud, fog, and edge computing with a focus on resource management. Consequently, we provide an analysis of solutions to these challenges from the existing literature. To this end, we identify and analyze 16 different resource management solutions and derive a taxonomy to evaluate them effectively. One of our key contributions refers to the classification and evaluation framework of resource management techniques. Another contribution is the analysis and discussion about the suitability of algorithms concerning a particular solution paradigm (i.e., cloud–fog, fog–edge, fog-only, and cloud-only solution). To make the functionality of the reviewed resource management techniques more explicit and present it in more detail, we provide a classification of the features given by the algorithms.

The remainder of this paper is organized as described below. Section 2 outlines the methodology that we follow in this research. In Section 3, we present architectural overviews on cloud, fog, and edge computing and address background information on resource management techniques. Section 4 discusses challenges and limitations in the cloud, fog, and edge computing related to resource management. Section 5 provides an evaluation framework for applied algorithms in cloud, fog, and edge scenarios. Section 6 presents a classification overview of the suitability of algorithms concerning a solution paradigm. Section 7 gives a discussion and an outlook for the limitations of this study. Section 8 concludes the study.

## 2. Methodology

**Figure 1:**
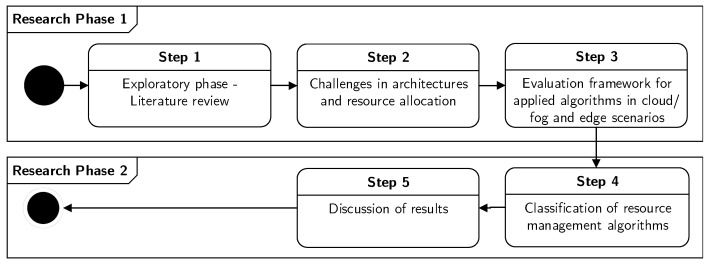
Research methodology followed throughout this paper.

We use the Design Science Research (DSR) methodology as discussed by Hevner [8] to structure the research in several steps (see Figure 1). The first phase is exploratory and discusses literature and challenges in the cloud, fog, and edge architectures with a focus on resource management. This represents the foundation for the development of the evaluation framework for applied algorithms in cloud/fog and edge scenarios, which is also completed in the first phase. The second phase includes the classification of resource management techniques, and a discussion of the findings.

The DSR is used as follows:

1. Research phase 1—Exploratory phase. This phase includes the DSR activities, which are necessary for the development of this research’s artifact (the evaluation overview), including the literature, knowledge base, and research theory.
  (a) Collect research studies regarding the architectural overview for cloud, fog, and edge computing and research on existing resource management techniques.
  (b) Gather knowledge about challenges in architectures for cloud, fog, and edge computing.
  (c) Collect literature on algorithms applied for cloud, fog, and edge scenarios.
2. Research phase 2—Classification and discussion. This phase includes the design and development of the second artifact (classification of the resource management techniques).
  (a) Overview existing literature for attributes related to resource management.
  (b) Classify and compare the literature.
  (c) Examine which research challenges are addressed by the articles.

## 3. Background and Related Work

This section first introduces a high-level architectural study of cloud, fog, and edge computing. The section proceeds with discussing the role of resource management techniques for such architectures. Finally, we discuss some architectural overviews of cloud, fog, and edge computing applied to particular application domains.

### 3.1. High-Level Architectural Overview

**Figure 2:**
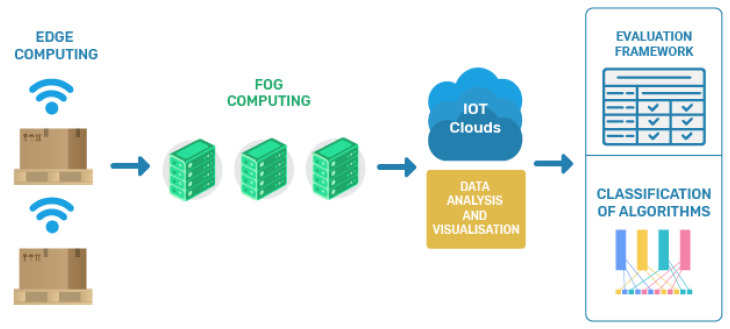
An example of cloud/fog/edge architecture for a smart pallet case study.

Figure 2 presents a high-level architectural design of a typical IoT infrastructure including cloud, fog, and edge infrastructures, which can be applied in a smart pallet logistics case study. The architecture consists of a cloud network as the top layer, a fog network as the middle layer, and an edge layer as the bottom layer.

The concept of cloud computing is about enabling anything as a service such that services can be merged, shared, and monitored with minimum involvement [9]. Users can access services in a ubiquitous manner, through the network, and on request. There is a certain amount of time that is needed to accomplish the communication between the cloud and the existing IoT devices, which will be automatically added to the processing time. The accumulated time is captured by the cloud servers and it contributes towards the increase of a system’s latency. Furthermore, this motivates the appearance of drastic effects on power and energy consumption [10]. As a result of the caused high latency, there can be indications of degradation in the Quality-of-Service (QoS) and Quality-of-Experience (QoE). Additionally, this will influence the reliability level of the system and generate delays in communication, capacity reduction, and excessive energy consumption. Some of the desired features for IoT infrastructures include modest latency, low response time, location awareness, low energy consumption, and portability support.

To accommodate some of these features, the computational paradigm fog computing was proposed [11]. In fog computing, data processing tasks are offloaded onto numerous middle-ware devices present in the network as a middle layer between the cloud and the end IoT devices. Each fog device is capable of processing the data that are being captured. This way, the overall latency is reduced, as the processing, happening locally, can lead to faster utilization, also locally, of the knowledge gathered. Fog computing represents the idea of broadening the cloud where the “things” are enhancing the application performance by removing the information processing within the cloud, and also by diminishing the bandwidth utilization in the network [12]. It has appeared as a promising technology that transports cloud applications in closer proximity to physical IoT devices. A fog node can also be seen as a mini-cloud, which is located near the edge layer of the network, and thus near the IoT devices connected to it [13]. A fog server represents a virtualized equipment, which contains on-board storage, computing, and communication capabilities. These features are meaningful when supporting the IoT application execution. Fog computing has been designed to deliver the following three core contributions: (1) diminish latency as the data are analyzed close to its sources from where it is initially gathered; (2) stabilizing network traffic, which is enabled by offloading gigabytes of network traffic from the core network connecting to the cloud; and (3) privacy and security support, which is enabled through proximity-by storing sensitive data in the nearby computer and network systems [12].

In edge computing, data processing is offloaded onto the edge devices [14]. Edge computing pushes the position of applications, services, and data to be close to the sources where such services are requested. In particular, the edge devices can be ’exploited’ by fog computing nodes to handle some of the calculations, storage, and transmissions locally. Edge computing technologies are commonly deployed on single devices.

A limitation of using solely cloud computing is that a centralized cloud computing concept may not be sufficient for data processing and analyzing the vast amount of data gathered from IoT devices. One cause can be the (massive) data transfer which results in limited network performance. Edge computing is typically about transferring computing tasks from a centralized cloud to the edge layer (near the IoT devices). As a result, the transferred data are typically already pre-processed and much more compact than raw information [14].

The design of efficient allocation mechanisms for processing data among resources spread within various layers can be challenging. Especially in (near) real-time scenarios, a decision needs to be made quickly. Consider the example of having two data processing types: batch and stream data processing. Processing (big) batch data may happen (mostly) in the cloud, while most of the stream data processing may be more suitable for being distributed to fog or edge nodes. Depending on the design, a small set of stream data may also need further processing on the cloud. Likewise, some pre-processing might also be necessary at an edge node before transferring data to higher layers. System designs are vital for maximizing the potential of both computing paradigms effectively in real-time environments.

### 3.2. An Example of Resource Allocation in a Cloud/Fog System

Resource allocation strategies in cloud/fog/edge systems are responsible for assigning accessible resources to the system users [15]. It can be a challenge to assign resources efficiently to applications and their end users/consumers [16].

**Figure 3:**
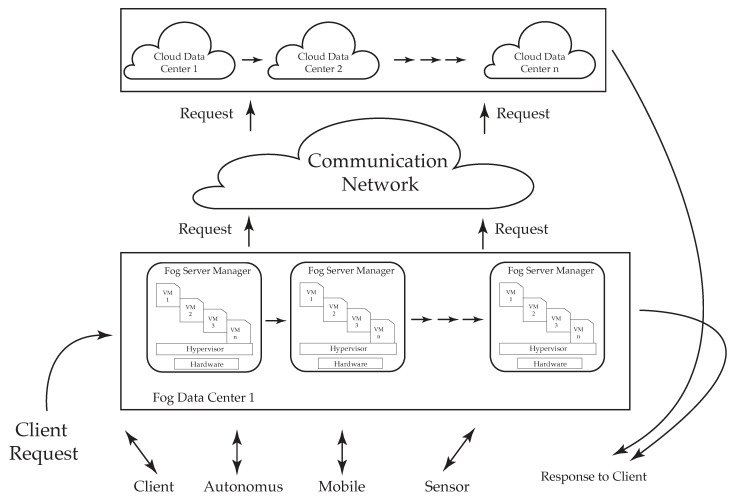
Three-layer architecture [16].

To give an example, consider the design model of [16]. This model administers the resource allocation in a fog environment (see the representation in Figure 3). The cloud–fog environment model is composed of three layers: a client layer, a fog layer, and a cloud layer. First, a solution for resource management is realized in the client and fog layers to accomplish the requirement of resources for clients. If there is no/limited availability of resources in the fog layer, then the request is passed to the cloud layer. The main functional components of this model are as follows:

- The fog server manager employs all the available processors to the client.
- Virtual machines (VMs) operate inquiries for the fog data server, process them, and then deliver the results to the fog server manager.
- Fog servers contain one fog server manager and virtual machines to conduct requests by using a ’server virtualization technique’.

### 3.3. Some Application Domains

#### 3.3.1. An Architecture Based on Cloud, Fog, and Edge Computing Paradigms in Real-Time Internets-of-EveryThings

According to Seal and Mukherjee [17], there are definite tiers of a universal fog computing architecture. Tier 1 depicts the ’Edge Tier’, which consists of multiple Terminal Nodes (TNs). TNs represent mobile and smart nodes that are capable of detecting various location parameters and then transmitting them to the upper layer. Tier 2 is known as the middle layer or the ’Fog Layer’. This layer is composed of smart devices: routers, gateways, switches, and access points that can contribute to data computation, data storage, routing, and packet delivery. Tier 3 is known as the cloud computing layer, which contains personal computers and servers.

Virtual Clusters (VCs) are defined as location-based parameters, which are composed of IoE devices often known as TNs. In this specific case, the role of TNs is to examine their environment and then transfer the data to the fog layer. Each Fog Instance (FI) monitors its own VC. The fog computing architecture can be further categorized into two sub-components: (a) the fog abstraction layer; and (b) the fog orchestration layer [17]. The first one deals with the management of fog resources, virtualization support, and configures tenant privacy, while the second layer contains the fog properties. Some of the fog properties are: heterogeneity, edge location, geographical distribution, support for mobility, real-time interactions, and interoperability. The fog orchestration layer consists of a software agent known as foglet, dedicated to monitoring the condition of the terminal devices. A decentralized database is used for scalability and fault tolerance, and the service orchestration module’s role is to be responsible for the policy-based routing of application requests. The orchestration module also needs to decide whether it will transmit to cloud centers.

The fog devices’ utilization is for limited semi-permanent storage, which facilitates provisional data storage and handles applications that are sensitive to latency. The cloud is accountable for the storage of large data chunks within its data centers. Those data centers typically contain massive computational abilities. The fog layer enables the cloud to be accessed and applied in an efficient and controlled manner.

#### 3.3.2. An Architecture for Smart Manufacturing Based on Cloud, Fog, and Edge Paradigm

Cloud computing reinforces ubiquitous and on-demand network access to a distributing pool of computing resources (e.g., processing and storage facilities, appliances, services, etc.). By using virtualization technology, cloud computing shelters the diversification of basic devices and provides different services in a transparent way to the users, including IaaS (Infrastructure-as-a-Service), PaaS (Platform-as-a-Service), and SaaS (Software-as-a-Service). Because of the expansion of various access devices, cloud computing can encounter obstacles in bandwidth, latency, network unavailability, privacy, and security. Fog computing is viewed as an expansion of cloud computing to the edge network, conducting services (e.g., computation, storage, and network) in close proximity to the end-user devices (e.g., network routers), instead of transferring data to the cloud [18]. In a fog computing concept, data storage and processing largely depend on local devices, rather than on a cloud system.

Complementary to fog computing, edge computing grants computation to be completed at the edge of the network and an approaching environment to the data sources [18]. The crucial divergence between fog and edge computing is that fog depends on the interconnection amid nodes, while edge computing operates in the segregated edge nodes.

Edge computing administers services nearby the data sources to meet the critical requirements on privacy, security, agile association, and real-time optimization [18]. In pursuance of enlarging the application of smart manufacturing, by utilizing the cloud and administering future aspects of smart solution applications, a reference architecture based on cloud/fog/edge computing for smart manufacturing has been recommended.

### 3.4. Techniques for Handling Resource Management

There are many articles discussing mechanisms for handling resource management (e.g., allocation, provisioning, workload balance, and task scheduling). Specific goals relate to reduction of the overall energy consumption, the latency, or the overall communication costs. This section presents a concise literature overview on different techniques for handling resource management in cloud/fog and edge computing. The metrics under which we evaluate the reviewed research are algorithm classification type, deployment scenario, resource management criteria (resource allocation, resource provisioning, workload balance, and task scheduling), QoS, energy management, and environment. In the appointed evaluation, we focus on analyzing 16 state-of-the-art methods.

In [19], the authors focused on resource management which is done in the fog layer, aiming to minimize latency and enhance reliability. There is a consumer layer where its users can accomplish their specific current demands via fog and cloud. Requests per hour, response time, and processing time parameters are considered by using the round-robin algorithm, equally spread execution algorithm, and a proposed algorithm. Their focus is on considering a fog and a cloud environment together for resource optimization. The authors implemented the Fog-2-Cloud framework for the management of customers’ requirements by utilizing six fog nodes and twelve MicroGrids in residential buildings. Fog servers helped in storing consumers’ private data. Their used performance parameters were response time, requests per hour, and computing time that can be improved by using the Shortest Job First (SJF) algorithm. This algorithm is compared with other techniques, Round Robin (RR) and Equally Spread Current Execution (ESCE), which outperformed the other two algorithms.

According to da Silva and d. Fonseca [20], fog and cloud can cooperate to advance their service distribution to the clients. This study is about a Gaussian Process Regression for Fog–Cloud Allocation (GPRFCA). It describes a mechanism that chooses where to allocate tasks based on the specific application requirements. The infrastructure is composed of a fog and cloud layer. The GPRFCA technique [20] decides where to appoint an assignment that needs to be computed while considering the availability of resources and latency costs. To advance the utilization of fog resources, GPRFCA is employed to predict the arrival of future requests based on the historical information. Such a prediction can support resource provisioning to future requests. That stands especially for real-time application requests which can only be processed within the fog. A simulation was performed, and its results represent that the given solution stabilizes the assignments between fog and cloud and the trade-off among latency, blocking, and energy consumption.

Fog-based computing and storage offloading for data synchronization in IoT create a large amount of data, which is partly due to the increase in IoT devices that are connected [21]. If, at a later stage, IoT devices transmit data to the cloud, then the data privacy can become a challenge. To address this, Wang et al. [21] proposed an architecture for data synchronization based on fog computing. It is achieved via offloading computing parts and storage work towards the fog servers and then data privacy can be better guaranteed. Additionally, a differential coordination founded on fog computing is recommended. The benefits of their composed architecture are: (a) data chunks can be stored in the fog server for enabling security; (b) the fog server facilitates the computation offloading and storage, which formerly belonged to the cloud and user’s devices; and (c) the transmission overload is minimized.

In [22], a method named Dynamic Resource Allocation Method (DRAM) is presented. This method relies upon static resource allocation and dynamic resource scheduling to achieve dynamic load balancing. Agarwal et al. [16] presented the Efficient Resource Allocation (ERA) method, which minimizes the response time and maximizes the throughput of resources. In [23], the authors discussed a method in which the resources are allocated according to a different priority. Taneja and Davy [24] proposed an iterative algorithm to reduce latency and energy consumption. Section 5 provides more details about the resource management techniques.

### 3.5. Related Work

Building further on the work discussed in [6], we provide an evaluation framework for resource management techniques applied in cloud/fog and edge computing scenarios. Naha et al. [6] provided a summary of research on resource allocation and scheduling only in the fog. It can be concluded that many articles addressed mainly the role of resource allocation in the fog environment. However, further investigation on QoS, load balancing, and energy efficiency needs to be considered [6]. There are several identified limitations regarding the use of fog computing. One challenge refers to the synthetic work done regarding the validation of methods [6]. Another challenge refers to the presentation of only cloud-based simulations, which are not completely suitable for the fog computing concept (which are typically dynamic environments).

Bendechache et al. [25] presented some research articles focused on resource allocation. Some of the explored resource management metrics were divided into two sections: resource provisioning and resource scheduling. Their provisioning metrics are detection, selection, and mapping, whereas the scheduling metrics are allocation, monitoring, and load-balancing. Additionally, several variables or Key Performance Indicators (KPIs) were investigated such as scalability, latency, VM placement, failure rates, accuracy, resource utilization, energy consumption, cost, efficiency, Service Level Agreement (SLA), and QoS. The contribution of this research survey is quite detailed, but it presents limited research articles that are focused on cloud, fog, and edge computing. Therefore, this represents a motivation to study resource management techniques for cloud, fog, and edge computing.

In [26], three types of taxonomies are demonstrated: (i) a classification of performance metrics for evaluating cloud, fog, and edge computing; (ii) metrics based on cloud models; and (iii) classification of identified metrics based on a concept known as MAPE-K. Based on the collected literature, the authors identified that the common performance metrics for cloud, fog, and edge computing include throughput, network congestion, fault-tolerance, statistical analysis measurements, scalability, cost/profit, and SLA violation. The taxonomy of metrics based on cloud models suggests the use of the following groups: private, public, hybrid, single-provider, multi-provider, and federated. According to a MAPE-K loop, there are four categories of parameters, including monitoring, analyzing, planning, and executing. Their results represent a mapping between the proposed taxonomy and existing literature on the cloud, fog, and edge computing paradigm. However, the study could be further extended by providing a proper detailed list of proposed solutions from the reviewed literature, and respectively their classification.

Ghobaei-Arani et al. [27] provided a taxonomy of resource management approaches in fog computing. The taxonomy considers the following categories: resource provisioning, application placement, resource scheduling, task offloading, load balancing, and resource allocation. They focused on structuring the literature according to resource management approaches. For each resource management approach, they provided details about the case study, utilized technique, used performance metric, evaluation tool, advantages, and weaknesses. Overall, this study provided knowledge about existing articles for each resource management approach, but only considering fog computing, while it would be also interesting to include edge computing. Additionally, the article only addresses the solution approaches in an exploratory manner. In other words, the research work represents an analytical examination and discussion on existing studies about resource management.

Lastly, Salaht et al. [28] delivered a list of optimization metrics to address resource management and service placement problems. The considered metrics are latency, resource utilization, cost, energy consumption, quality of experience, congestion ratio, and blocking probability. Based on the findings of Salaht et al. [28], further research work should be done on challenges regarding service placement problems, optimization strategies, and evaluation environments.

## 4. Challenges in Resource Allocation for Cloud, Fog, and Edge Computing

There exist several challenges regarding cloud, fog, and edge architectures, such as the deployment of 5G, serverless computing, resource allocation, optimization, energy consumption, data management, applying federation concepts to fog computing, trust models, business and service models, mobility, and industrial IoT [29]. A challenge in 5G includes realizing the concept of network shredding to backup a service collection with certain performance requirements requests. Some of them are: resource management throughout, fog nodes, wireless, optical packets, and cloud domains [29]. Recent developments in network virtualization grant guidelines for network shredding, but they do not provide a unified and general collection of resources over various domains. Based on the reviewed literature, we present the challenges in Table 1.

In terms of *serverless computing* [29], to achieve micro-services management through the cloud/fog/edge hierarchy, there are challenges regarding the flow of services among cloud, fog, and edge computing devices. The automatic administration of the micro-services must audit the deployment location and context; in addition, the resource constraints that may exist in the fog need to be taken into account. Additionally, the diversity of the system across an IoT cloud–fog ecosystem can be challenging for the deployment of micro-services and reconfiguration.

Furthermore, the network topology can be expected to change regularly due to devices mobility and changing application requirements. The high levels of heterogeneity in IoT devices and the variability of the environment call for active and dynamic system management based upon multi-criteria *resource allocation* [29]. Resource management systems and multi-criteria schedulers may instantly enhance resource allocation in terms of handling dynamic behaviours. That can be challenging since the number of variables can largely expand the search area and that can consequently lead to long scheduler execution times.

A substantial demanding route for prospect research is in diminishing *energy consumption* [29] where the target should be researching the aspect and significance of data in the cloud/fog/edge ecosystem, onward with the definition of ‘economical data management’. The objective behind this is monitoring in detail the implication of various data types and whether the data are required most of the time.

In recent years, there is an expansion in production and use of data, and that accomplished a few remarkable rates. Concerning *data management and locality* [29] in IoT cloud–fog computing systems, accessibility problems have to be considered. Computing systems consist of several networking technologies, such as mobile, wireless, or wired. When the resources are centralized within the cloud, certain networking challenges, such as availability, scalability, and interoperability, might be partially addressed. However, some of the innovative problems (e.g., network bottlenecks and latency) can be addressed by using fog and edge computing. A particular challenge is how to quantify the trade-off among data distribution and services at the fog or cloud layers. One way towards approaching this issue is via smart service placement. More specifically, this can be done by data locality, which is achieved by placing the needed services closer to the data that they administer. Suitable candidates, according to Bittencourt et al. [29], are applications that do not need high computation power and are capable of analyzing large data volume.

In terms of *orchestration in fog for IoT* [29], privacy requires to be tackled according to the European Union General Data Protection Regulation (GDPR) and other similar regulations imposed all around the world. Privacy regulations are important because, when fog nodes are placed close to the end-users, one may attempt to gather, process, and store data, and that can violate users’ privacy. The performance of fog orchestration for the IoT deals with several challenges, related to 5G networks such as an increase in density of devices combined with latency and reliability requirements of demanding applications along with the mobility of nodes, which boost important problems concerning the system monitoring, and that is significant for proper resource management. Other fundamental aspects that directly impact the performance of (dynamic) fog orchestration are component selection and placement, which need to be additionally investigated in the future, as well as research on efficient techniques to prevent (minimize or stop) the overloading and avoid delays.

Another challenge is *business and service models*. The fog can be deployed as a hybrid cloud, where specific local resources can be extended with resources from the cloud. Additionally, when different stakeholders are incorporated in a specific hierarchy from IoT to the cloud, this can create a scenario in which different elements of the overall systems are owned or managed by completely unrelated entities or stakeholders, e.g., IoT devices can be owned by the state, while fog nodes are owned by a cloud company. It is challenging to determine how IoT services can be combined with services from fog and cloud computing, and then how they can be monitored and administered when many players at different levels are participating.

In [30], the challenges with IoT appliances in cloud, fog, and edge computing are related to replying to resource requirements and *load balancing*. In this article, load balancing is considered as one of the meaningful strategies to accomplish efficient usage of resources and reduce or avoid congestion. Therefore, it is a distinguishing challenge to obtain load balance for the processing nodes in a fog environment all along with an IoT application execution. According to [20], the determined challenge was regarding minimizing latency as well as balancing the workload to reduce energy consumption.

The limitations considered in [21] are related to cloud computing and *cloud-based synchronization*, which is a particular core service in the cloud computing area. The IoT devices synchronize most of the data to the cloud. There are two challenges in this specific scheme referring to *security and efficiency issues*. The security issues regarding cloud storage revolve around the following aspects: *integrity, privacy, and availability of data*. The selected established security threats are data exposure, data deficiency, malicious user handling, wrong use of cloud computing and its services, and possibly session stealing during data accessing. Problems such as connection cost and latency between the cloud system and edge layer devices are not tolerable in detention-sensitive applications. While there are, for example, some synchronization tools such as MicrosoftActiveSync and Botkinds AllwaySync, the drawback is that they regularly transmit an entire system file even when there is a small change occurrence. This coordination type may cause redundant communication and latency issues, where users frequently modify the data. It can be concluded that traditional coordination among cloud and IoT devices has certain disadvantages such as when the IoT devices fail to secure confidential data, and/or when common data changes cause high data and communication redundancy.

In [22], limitations of resource requirements and load balancing for IoT appliances in cloud, fog, and edge computing are presented. Load balancing is an important factor that is valuable to increase resource efficiency by avoiding bottlenecks, overload, and low load situations. Accordingly, it is an obstacle to accomplish load balance for the computing nodes in a fog environment at the same time as the IoT application execution occurs. According to Taneja and Davy [24], cloud computing offers many assets, but with expansion in more ubiquitous mobile sensing devices coupled with technological upgrades, the imminent IoT ecosystem demands the computing network architecture of the cloud. A few of the requirements that need to be met are *dynamic scalability, efficient-in-network processing, and latency-sensitive communication*; these are the requirements for IoT application which drove the evolution of fog computing.

## 5. Evaluation Framework for Resource Management Algorithms in Cloud/Fog and Edge Scenarios

Table 2 shows an overview of selected techniques used in the reviewed literature about resource management in cloud/fog and edge-based scenarios. The resource management algorithms are summarised in the table and evaluated according to several metrics that are discussed below. Resource management is about achieving coordination of resources that is highlighted by supervision (management) actions and performed by service providers and users [31]. It considers the resource allocation process from resource providers to the users. The algorithms discussed in the following subsections employ different resource management metrics which are examined as well and can be used for further evaluation.

### 5.1. Resource Allocation

Resource allocation represents a technique that is used to optimize the utilization of resources and reduce the required costs for processing [32]. Fulfillment time of a task is an important aspect that should be considered since it can impact the completion of resource allocation [33]. As indicated in Table 2, RR, ESCE, SJF, GPRFCA, ERA, Priority-based Resource Allocation algorithm (PBSA), and Feedback-Based Optimized Fuzzy Scheduling algorithm (FOFSA) use resource allocation techniques.

### 5.2. Workload Balance

Workload balancing is an important factor used to manage energy effectiveness and also avoid congestion, low-load resource management, and overload. Currently, this represents a challenge for the processing nodes, which are placed in the fog environment. For instance, in [34], a workload balancing algorithm is proposed for fog computing, aiming to reduce the data flow latency in the transmission procedures by connecting IoT devices to the appropriate base stations (BSs). The article discusses several workload balancing algorithms from the literature: RR, SJF, ESCE, GPRFCA, DRAM, ERA, PBSA, FOFSA, Hill Climbing algorithm (HCLB), Efficient Load Balancing algorithm (ELBA min-min), and Tabu Search algorithm.

### 5.3. Resource Provisioning

Resource provisioning represents an approach (solution) that shows how to administer requests for tasks and data among fog nodes [35]. Resource provisioning is a further step in resource allocation. As discussed above, resource allocation deals with just assigning a set of resources to a task, while resource provisioning deals with the activation of the allocated resources. Remote Sync Differential Algorithm (RSYNC), Fog Sync Differential Algorithm (FSYNC), Reed–Solomon Fog Sync (RS-FSYNC), ERA, and Energy-aware Cloud Offloading (ECFO) are the algorithms that deal with resource provisioning.

### 5.4. Task Scheduling

To manage a large set of tasks that are working together and are dependent on a certain set of resources, task scheduling algorithms have been proposed to define a schedule to service tasks to avoid conditions such as deadlocks [36]. Table 2 shows a few algorithms that manage resources based on task scheduling: RR, SJF, ESCE, GPRFCA, DRAM, PBSA, FOFSA, ELBA, Tabu, and ECFO.

## 6. Classification of Resource Management Algorithms Applied in Cloud/Fog and Edge Scenarios

**Figure 4:**
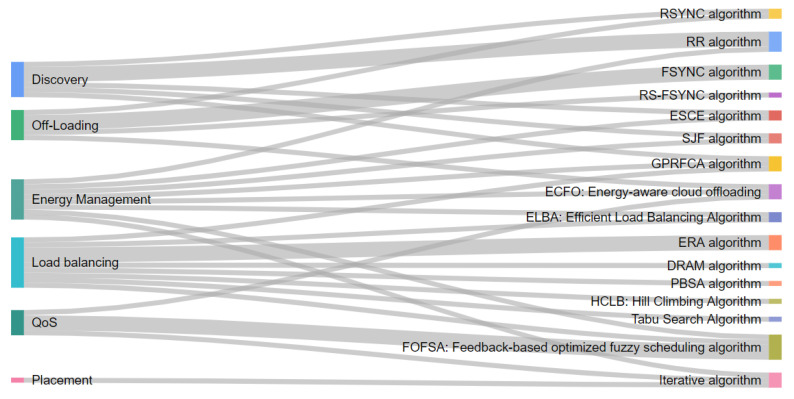
Classification of resource management techniques.

To compare the various state-of-the-art algorithms presented in several papers, inspired by Hong and Varghese [42], we classify the selected algorithms into six categories. Classification helps in terms of the identification of existing solutions and understanding their diversity as well. It can support researchers and practitioners in the process of learning about different algorithmic solutions to understand their features, differences, and similarities. The reviewed solutions consider how resources are handled among cloud, fog, and edge devices. In this paper, we briefly overview these 16 algorithms. They represent the basis for building the evaluation framework (Table 2), which is the foundation of this paper, and the emerging classification of the algorithms are presented in Figure 4. We created this classification to address the key contributions in the area of resource management.

### 6.1. Discovery

Discovery is used to find available resources from the cloud, fog, or edge layers, based on workload requirements, to identify where they can be deployed efficiently. Fog servers have to use as many resources as desirable through accepting a high volume of tasks as possible. A manner of doing this is by using a manager or master entity that has an overall view of the resources. Afterward, based on the workload’s requirements, it can allocate resources properly among fog and cloud layers. According to Hong and Varghese [42], in the edge/fog computing concept, the discovery algorithms stand for determining resources in the edge network that can be employed for further distributed processing.

For example, according to Javaid et al. [19], the algorithms RR, ESCE, and SJF belong in this category. GPRFCA and RSYNC belong in this group as well.

- RR—Round Robin Algorithm: According to the authors of [19], the RR algorithm for cloud computing has been adopted on the basis of defining time schedules. The scheduler creates certain specifics of VMs in an assignment table. Then, it assigns jobs that are received for data centers (DCs) to a set of VMs. Initially, a VM is initialized with an ID of a current VM variable and then the demanded job is mapped with the current VM variable.
- ESCE—Equally Spread Current Execution: The ESCE algorithm enforces the spread spectrum approach and collaborates with a large number of active duties on VMs at any specific time segment [19]. By using ESCE, the scheduler can register the VMs’ assignment table, and then keep up a list of VMs’ IDs and their operating tasks on any VM. Once the task is performed, at any specific time interval, the VM table can be changed. In the beginning, the active task count is 0; on the occurrence of a new job, the scheduler determines the VM having the minimum task count. If many tasks are assigned to many VMs that are with the minimum count, then the first VM will be selected for the task processing.
- SJF—Shortest Job First: The SJF algorithm executes tasks by labeling the task size as a priority, and the priority is further controlled by the size of consumers’ requests [19]. SJF can allocate tasks to VMs based on their fogs, the priority of distances, and size. The scheduler can be used to distribute the job on VMs based on the spread spectrum approach. SJF schedules the jobs by enabling minimum completion time, higher efficiency, and minimum turn-around time.
- GPRFCA—Gaussian Process Regression for Fog–Cloud Allocation: The GPRFCA mechanism is used to discover predictions to govern work activities on fog nodes while reducing latency [20]; as such, it belongs to the discovery group. Generally, it investigates the history of formerly sent requests for future arrivals’ predictions of VMs, which are by rigorous latency demands [20]. By adopting these predictions, this technique can store the required resources within the fog nodes for future requests. Consequently, they should be completed within the fog layer itself, and then tasks that are not vulnerable to delays are assigned in the cloud. This leads towards an increase in fog nodes’ utilization [20].
  CPU and RAM are important assignable resources for this mechanism [20]. The algorithm starts with the calculation of the number of VMs which can be still executed by the fog node (this is done by taking into consideration CPU and RAM). Furthermore, the Gaussian Process regression is then called (Line 4) to predict the VMs number, ***future VMs***, which should be incorporated also in the fog, but at the next interval.
- RSYNC—Remote Sync Differential Algorithm: RSYNC is one of the first algorithms to face the problem of complete synchronization whenever an update (change in file) is performed [21]. As the name implies, this differential algorithm is used to transmit only that particular part of the data that experiences an update. Since every instance of synchronization sends a small piece of information, the communication cost and latency decreases when compared with previous algorithms. Nevertheless, RSYNC is more suitable for establishing a communication path between IoT devices and the cloud layer. Although it sends only the updated data, it still needs to send a synchronization request every time that IoT device does an update.

### 6.2. Off-Loading

Off-loading is accountable for the resource provisioning tasks. It concentrates on storage provisioning instead of computation. It determines where data should be stored to lower transmission expense and the delay between the cloud computing layer and IoT (edge) devices [41]. Following Wang et al. [21], we identify two main differential synchronization algorithms, RSYNC and FSYNC, since they are focused on where the storage is provisioned properly. FSYNC decreases the latency and the communication costs considerably due to the use of the fog layer and a specific defined threshold. The threshold refers to the number of trivial changes that can be saved in the fog.

- RSYNC: This algorithm is explained in the previous subsection.
- FSYNC—Fog Sync Differential Algorithm: The FSYNC algorithm deals with the RSYNC issue [21]. The issue refers to the case that there are many requests when the edge device is modified. During each request, new data are generated, which lead to the creation of additional load on the cloud server. It differentiates by adding two elements, a fog computing layer, and a threshold. It establishes a threshold, and then, when the IoT device updates, the algorithm will send only the part of the data that has changed to the fog layer. The difference is that there are no requests and data being sent to the cloud. Additionally, only when the threshold is reached the fog servers will send a complete synchronization of the data. Otherwise, the following updates will be done between fog servers and IoT devices.
- RS-FSYNC Differential Algorithm: RS-FSYNC is a (Reed–Solomon Fog Sync) differential algorithm [21]. By applying the Reed–Solomon code, the security of the user’s data can be enhanced. The Reed–Solomon code is included in the FSYNC algorithm. Additionally, it uses an advantage from the storage capacity of the fog server to handle an encryption problem. Furthermore, it represents a variant of erasure code that was used within the distributed storage field. The objective is to revise errors created by the redundant data, which is generated by the original data.
- ECFO—Energy-aware cloud offloading: The energy expenditure of a local device can be accordingly diminished by offloading computational tasks to a remote device. Although supplementary transmission energy and communication latency may happen due to the appearance of data transmission between the remote device and local system [41], the specific challenge addressed by ECFO is how to distribute multiple tasks to and from multiple fog devices taking into account each device computational ability and the overall communication constraints [41]. To solve this problem, the ECFO algorithm tracks the bandwidth and schedules queues between devices to detect the energy consumption and provide an offloading decision. The process is dedicated to scheduling offloading activities into a two-phase deadline in order to dynamically adapt to changes in run-time network bandwidth. In the end, it also plans setbacks, which are caused by devices with multiple tasks.

### 6.3. Load-Balancing

Load-balancing distributes the workload to resources to make the operations more efficient by avoiding congestion, low load, and overload [22]. The considered algorithms based on load-balancing are DRAM [22], ERA [16], PBSA [23], GPRFCA, FOFSA, HCLB, ELBA, and Tabu Search algorithm.

- DRAM—Dynamic Resource Allocation Method: DRAM [22] is a dynamic resource allocation method that consists of the following steps:
  – Fog service partition: This is pre-processing in which the fog services can be categorized according to the resource requirement of each node type [22].
  – Spare space detection: To decide whether a node is portable to accommodate a fog service, identifying the extra space of all processing nodes is needed [22].
  – Static resource allocation for the fog service subset: For services within the fog that belong to the same subset of services, the appropriate processing nodes are selected to accommodate them [22]. When allocation starts, the node with the lowest extra space is selected.
  – Load-balance global resource allocation: The dynamic resource allocation strategy is executed to achieve load balance [22].
- ERA—Efficient Resource Allocation Algorithm: The ERA algorithm in [16] was designed to achieve effective resource allocation in the fog layer. The client makes a request and this request can be accepted only by the fog layer. If the fog does not process the request within a given time frame, then the process is transmitted towards the cloud [16]. With this method, the response period is diminished and the throughput is increased.
- PBSA—Priority based Resource Allocation Algorithm: In PBSA [23], batches of user’s requirements are created according to the type of the task, the processing amount, and the time that the clients need [23]. If the specific resources that the user needs are not there, then the client needs to wait until they become available. If two identical requirements have a particular request with the same priority, then the method of ’first comes, first served’ is used.
- GPRFCA: The GPRFCA algorithm belongs to this category as well.
- FOFSA—Feedback-Based Optimized Fuzzy Scheduling Algorithm: The Feedback-Based Optimized Fuzzy Scheduling Algorithm (FOFSA) is proposed in [37]. FOFSA works with two procedures: multilevel queue scheduling and multilevel feedback queues. The job activities are enrolled in different levels of queues. The queues are managed based on the concept of ’first come, first served’. The job activities can be appointed to resources per specific priority. If the job activity is not assigned to a particular resource, then the job is simply removed from the waiting sequence. A task’s priority can be decided by the fuzzy inference system procedure presented in [37]. Additionally, an architecture of the fuzzy-based scheduling is introduced in [37]. The proposed methodology was tested with iFogSim and analyzed with different existing dynamic algorithms. It was justified by the fact that it contains an effective scheduling strategy and upgrades the QoS parameters. The suggested methodology achieved a reduction in power utilization and enforcement time.
- HCLB—Hill Climbing Algorithm: HCLB algorithm is defined as a mathematical optimization technique that is used for searching and monitoring the loads among VMs [38]. This technique is established on a random solution to discover accessible VMs. The goal of the algorithm is to find a solution to the problem of discovering accessible VMs, and the searching loop executes only when the appropriate solution is found [38]. When the nearest VM is detected, the loop is increased in HCLB [38]. Then, the best VM is selected, and a request is assigned to it for further processing.
- ELBA—Efficient Load Balancing Algorithm: The min-min algorithm is implemented in the fog where fog nodes are divided in clusters and the algorithm determines the task which has minimum enforcement time and appoints it a particular node. That node is able to process it in a faster manner [39]. When a cluster is busy, the controller node inspects neighborhood clusters that contain ’inactive’ fog nodes and sends activity to the node which presents lowest latency. Afterwards, the cluster shall send the activity with the favorable latency. If the cluster with ’inactive’ fog node is located far away, then the particular task should be instantly sent to a cloud system for further processing. It could be effective to process the activity in the cloud or, instead, leave it to have a delay due to pre-processing at the fog nodes. In another situation, where two or more neighboring ’inactive’ nodes are accessible, the node with the smallest latency can transmit the job activity [39]. Two factors need to be deliberated to calculate latency: one refers to the number of stand-by requests that need to be supplied in the clusters and the other refers to the inactive node’s distance from the task originator. Calculation of the lowest distance between the source node and a fog node or a cloud data center can be determined by using Equation (1) [39]. N represents minimum latency, S is the source from where a particular activity is re-transmitted, C is the nearest cloud data center, and n depicts the number of fog nodes.
  (1) $$N=min\left[\left[d\left(s,c\right)\right],min\sum_{i=1}^{n}\left[d\left(s,ni\right)\right]\right]$$
- Tabu Search Algorithm: Tabu search is used to determine an optimal solution regarding the distribution of tasks between nodes that belong in the cloud and fog layers. It is done by utilizing search which frequently moves towards an improved solution every time [40]. The searching process will be terminated the moment a stopping condition is detected. Optimal load balancing is one of the biggest concerns in fog computing. To accomplish optimal load balancing, [40] used Tabu search in fog computing for load balancing. In this study, a bi-objective cost function was considered to achieve online computations, where the initial one implies the computation cost of computing tasks in the fog nodes, and the second one supports it in the cloud.

### 6.4. Placement

Placement is used to determine the suitable resources to satisfy the required workload. The main purpose is to distribute the incoming computation tasks to the appropriate fog/edge resources.

- Iterative Algorithm based on resource placement: [24] proposed an iterative method that is based on resource deployment of IoT applications in a cloud–fog computing setting. This method is composed of three algorithms. The first algorithm sorts the network nodes and application modules according to their requirements and capacity (CPU, RAM, and network bandwidth). The second algorithm looks for an eligible network mode that meets the module’s requirement. The last algorithm is responsible for ensuring the requirement check, which is done by using the COMPARE function [24].

#### 6.4.1. QoS

We distinguish QoS as one of the classification categories of resource management techniques. Additionally, it can be used as a feature that may be used for further evaluation of the reviewed algorithms. When taking into consideration the use of cloud computing, as a solution concept, we should be aware that the data transfer between cloud and clients will contribute towards the increase in feedback latency [43]. This will lead to restrain the cloud service to provide quality of service to clients [43]. The QoS concept is defined in the ITU-T Recommendation E.800 and refers to the following [44]:

*“The collective effect of service performance, which determines the degree of user’s satisfaction of the service.”*

The QoS consists of a set of parameters that pertain to the traffic performance of the network, but, in addition to this, the QoS also includes additional concepts. Therefore, they can be summarised as:

- Service support performance
- Service operability performance
- Serviceability performance
- Service security performance

The following group of reviewed algorithms belong in this category: Iterative Algorithm based on resource placement, FOFSA, and ECFO.

#### 6.4.2. Energy Management

Enormous amount of energy savings can be obtained by taking into consideration energy consumption and energy management, which are associated with IoT and the cloud, fog, and edge paradigms [29]. Various methods can be used to address these concerns such as: (1) algorithms for energy-aware data transfer; (2) algorithms that limit the amount of data which is transferred within the network by utilizing certain criteria (thresholds); and (3) algorithms which exchange processing with communication, by using concrete objectives to achieve a balanced trade-off [29]. Based on the reviewed literature, energy management is selected as one of the classification categories for resource management techniques, to which we consider that the following algorithms belong: RR, SJF, ESCE, GPRFCA, Iterative algorithm based on resource placement, FOFSA, ELBA(min-min), and ECFO.

## 7. Discussion and Limitations

One of the key contributions of this paper is to provide an evaluation and classification overview of applied algorithms for resource management that address cloud/fog and edge environments. To support researchers in the further evaluation analysis process, they may initially need to understand the cloud/fog/edge architecture concept, and then learn about the potential challenges. In the end, researchers can finally explore in detail the existing resource management techniques that can address some of the potential challenges. Conforming to the conducted literature review, we identify a few solutions out of the 16 algorithms that can respond to some of the challenges, as shown in Table 3.

There exist certain challenges regarding resource allocation on a cloud/fog/edge network. When data are processed and then saved in a cloud system and if data centers are positioned far away from the devices, the complete process of data storage and processing may take a long time. Then, tasks need to be distributed in a manner that the entire network of devices inside a fog computing infrastructure can be completely utilized. If they are concentrated only in one particular area of the network, it will replicate a traditional cloud computing model which is not a desired factor. The distributed task allocation is focused on diminishing the average latency of service while lowering the overall quality loss.

**Figure 5:**
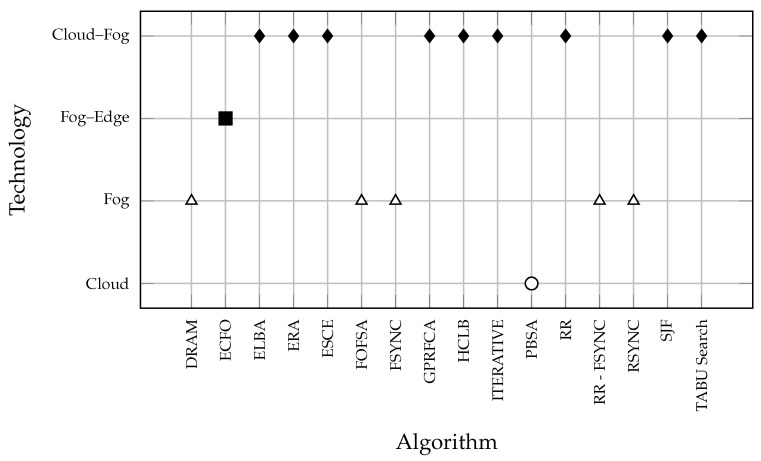
Reviewed algorithms per type of solution paradigm.

The analyzed algorithms are grouped per type of solution paradigm, as represented in Figure 5. It clearly illustrates which algorithms belong to a specific type of solution paradigm: cloud–fog, fog–edge, fog-based, or cloud-based. The majority of analyzed techniques (nine of them) belong to the cloud/fog paradigm, while five are only fog-based solutions, one is fog/edge type, and one technique is only a cloud-based solution. Additionally, this indicates that researchers and experts in IoT could focus on developing an algorithm that will address resource management challenges in the complete cloud/fog/edge paradigm.

Furthermore, this review proposes that algorithms can be classified according to their characteristics in six classes: discovery, load-balancing, off-loading, placement, QoS, and energy management. The discovery group finds available resources from either the cloud or fog layers based on specific workloads requirements, to identify where they can be deployed efficiently. The following algorithms belong to the discovery group: RR, ESCE, SJF, GPRFCA, and RSYNC algorithm. The offloading group is responsible for resource provisioning tasks, with the corresponding algorithms: RSYNC, FSYNC, RS-FSYNC, and ECFO algorithm. The load-balancing group handles the distribution of workload to resources. The algorithms which are considered in this group are DRAM, ERA, PBSA, GPRFCA, FOFSA, HCLB, ELBA (min-min), and Tabu Search Algorithm.

The placement group refers to finding the suitable resources to deploy the workload. Placing the incoming computation tasks to appropriate fog/edge resources is important. The only found algorithm that belongs to this group is the Iterative Algorithm based on resource placement.

Most of the algorithms were evaluated by using CloudAnalyst, SimCloud, Cloudlet tool, OMNET++, or iFogsim tools. Some studies in resource scheduling have experienced low scalability and proposed centralized topology in several case studies. One of the most important factors is scalability in resource management of fog computing, which should be improved in the scheduling scenarios. In addition, self-adaptive resource scheduling is one of the key issues in resource management of fog computing that few research studies have considered.

The provided evaluation of resource management techniques is limited to the features provided in Table 2. From a particular group/category of resource management techniques, we assume that ’the most appropriate’ responsive algorithm provides all the features. For instance, all discovery algorithms can be ’appropriate’ (except for RSYNC), since all these algorithms support the same metrics: resource allocation, workload balance, and task scheduling (refer to Table 2). From the reviewed load-balancing algorithms, the ’most’ responding algorithms to our criteria are FOFSA and ERA. FOFSA pillars resource allocation, workload balance, and task scheduling, while ERA supports resource allocation, workload balance, and resource provisioning. An offloading algorithm that meets most of the specified criteria is ECFO, which performs resource provisioning and task scheduling.

This evaluation framework can be extended by applying a multi-criteria decision-making method, which could help the readers in the decision process to select a resource management algorithm. One of the limitations of this paper is that it does not provide any experiment to test the application of the researched algorithms to verify their usability and competitiveness. Another limitation in this research is that we propose an evaluation framework for resource management techniques that can be applied in cloud/fog and edge environments, but there is not a proper comparison analysis of the indicated approaches.

## 8. Conclusions

The goal of this paper is to provide an evaluation framework and classification of different resource management techniques that can be applied in cloud/fog and edge scenarios. It is useful for cloud/fog/edge architects to have a concise representation of the various challenges in resource management.

Cloud, fog, and edge computing govern a paradigm that can offer a solution for IoT applications that are sensitive to delay. Besides, fog nodes usually have higher repository capacity and data processing, which can be used for improving performance and reducing cost communication and latency. To be able to evaluate the state-of-the-art algorithms used in multiple research articles, in this paper, we analyze algorithms that can be classified into six categories. Thereafter, we consider how resources can be handled among cloud, fog, and edge devices.

In future work, the focus can be on making an analysis and comparison (e.g., through simulations) between them rather than an evaluation overview. Furthermore, some of the reviewed algorithms can be used in the simulation of a cloud/fog/edge architecture suitable for a particular application domain (e.g., smart logistics). We suggest research on case studies, preferably from a variety of domains.

Additional research work can also be done in terms of investigating (new) algorithms that not only deal with resource management but also address other challenges in cloud/fog/edge computing environments. We also recommend further research on validating and extending the evaluation framework and classification method, for example by conducting a systematic literature search.

## Figures and Tables

**Table 1:** Challenges in architecture for cloud, fog, and edge computing.

Challenges	References
Serverless computing	[29]
Energy consumption	[29]
Data management and locality	[29]
Orchestration in fog for IoT	[29]
Business and service models	[29]
Load balancing	[30]
Security and efficiency issues	[21]
Data integrity and availability	[21]
Cloud-based synchronization	[21]
Dynamic scalability	[24]
Efficient network processing	[24]
Latency sensitivity	[24]

**Table 2:** Evaluation framework for applied algorithms in fog–cloud and edge scenarios.

Resource Management Techniques in Fog/Cloud Edge Scenarios										
**Author & Year**	**Algorithm**	**Deployment**	**Classification**	**Resource Management**				**Additional Classification**		**Environment**
				**Resource** **Allocation**	**Workload** **Balance**	**Resource** **Provisioning**	**Task** **Scheduling**	**QoS**	**Energy** **Management**	
Javaid, S. et al., (2018) [19]	RR	Simulation (Cloud Analyst)	Discovery	✓	✓		✓		✓	Cloud–Fog
Javaid, S. et al., (2018) [19]	ESCE	Simulation (Cloud Analyst)	Discovery	✓	✓		✓		✓	Cloud–Fog
Javaid, S. et al., (2018) [19]	SJF	Simulation (Cloud Analyst)	Discovery	✓	✓		✓		✓	Cloud–Fog
Da Silva, R.A.C. et al., (2018) [20]	GPRFCA	Simulation iFogSim [12]	Discovery & Load-balancing	✓	✓		✓		✓	Cloud–Fog
Wang, T. et al., (2019) [21]	RSYNC	Experiments in different conditions, two situations of synchronization	Discovery & Off-loading			✓				Fog
Wang, T. et al., (2019) [21]	FSYNC	Experiments in different conditions, two situations of synchronization	Off-loading			✓				Fog
Wang, T. et al., (2019) [21]	RS - FSYNC	Experiments in different conditions, two situations of synchronization	Off-loading			✓				Fog
Xu et al., (2018) [22]	DRAM	Evaluation done with three different types of computing nodes	Load-balancing		✓		✓			Fog
Agarwal et al., (2016) [16]	ERA	Simulation (Cloud Analyst)	Load-balancing	✓	✓	✓				Cloud–Fog
Savani et al., (2014) [23]	PBSA	Simulation (CloudSim 3.0.3)	Load-balancing	✓		✓				Cloud
Taneja et al., (2017) [24]	Iterative Algorithm	Evaluation done in three different topologies with different workloads	Placement					✓	✓	Cloud–Fog
Arunkumar et al., (2020) [37]	FOFSA	Simulation iFogSim	Load-balancing	✓	✓		✓	✓	✓	Fog
Chandak et al., (2018) [38]	HCLB	Simulation CloudAnalyst tool	Load-balancing		✓					Cloud–Fog
Manju et al., (2019) [39]	ELBA (min-min)	Simulation CloudAnalyst tool	Load-balancing		✓		✓		✓	Cloud–Fog
Téllez et al., (2018) [40]	Tabu Search	Simulation Cloudlet Tool	Load-balancing		✓		✓			Cloud–Fog
Jiang et al., (2019) [41]	ECFO	Cloud server and three Raspberry Pi3 devices	Off-loading			✓	✓	✓	✓	Fog–Edge

**Table 3:** Addressed challenges.

	Algorithm	References
Load Balancing [30]	GPRFCA	[20]
	ERA	[16]
	DRAM	[22]
	PBRA	[23]
	HCLB	[38]
	ELBA(min-min)	[39]
	Tabu Search	[40]
	FOFSA	[37]
Energy Consumption (Management) [29]	RR	[19]
	SJF	[19]
	ESCE	[19]
	Iterative algorithm	[24]
	FOFSA	[37]
	ELBA(min-min)	[39]
	ECFO	[41]
	GPRFCA	[20]
